# Diagnostic performance of alpha-fetoprotein, lens culinaris agglutinin-reactive alpha-fetoprotein, des-gamma carboxyprothrombin, and glypican-3 for the detection of hepatocellular carcinoma: a systematic review and meta-analysis protocol

**DOI:** 10.1186/2046-4053-2-37

**Published:** 2013-06-06

**Authors:** Ting-Shuo Huang, Yu-Chiau Shyu, Robin Turner, Huang-Yang Chen, Pei-Jer Chen

**Affiliations:** 1 Division of General Surgery, Department of Surgery, Chang Gung Memorial Hospital, Keelung, No.222, Mai-Chin Road, Keelung 20401, Taiwan; 2 Institute of Biopharmaceutical Sciences, National Yang-Ming University, No. 155, Sec.2, Linong Street, Taipei 112, Taiwan; 3 Department of Education and Research, Taipei City Hospital, No.145, Zhengzhou Road, Taipei 10341, Taiwan; 4 Screening and Test Evaluation Program, Sydney School of Public Health, Edward Ford Building (A27), The University of Sydney, Sydney, NSW 2006, Australia; 5 Graduate Institute of Clinical Medicine, National Taiwan University College of Medicine, No.7, Chung San South Road, Taipei 10002, Taiwan

**Keywords:** Hepatocellular carcinoma, Alpha-fetoprotein, Lens culinaris agglutinin-reactive alpha-fetoprotein, Des-gamma carboxyprothrombin, Glypican-3, Biomarker, Systematic review, Diagnosis

## Abstract

**Background:**

Diagnosis of early-stage hepatocellular carcinoma (HCC) followed by curative resection or liver transplantation offers the best chance for long-term patient survival. Clinically, ultrasonography has suboptimal sensitivity for detecting early-stage HCC. Several serological tests including alpha-fetoprotein (AFP), the ratio of lens culinaris agglutinin-reactive alpha-fetoprotein to total AFP (AFP-L3/AFP), des-gamma carboxyprothrombin (DCP), and glypican-3 (GPC-3) have been widely investigated as diagnostic biomarkers for early-stage HCC in at-risk populations. However, these tests are not recommended for routine HCC screening. Our objective is to determine the diagnostic performance of AFP, AFP-L3/AFP, DCP, and GPC-3 for the detection of HCC, particularly early-stage tumors meeting the Milan criteria.

**Methods/design:**

We will include cross-sectional studies that consecutively or randomly recruit target populations. We will search the Cochrane Library, Medline, Embase, Science Citation Index, and the Chinese National Knowledge Infrastructure. We will also search the MEDION and ARIF databases to identify diagnostic systematic reviews that include primary studies. Reference lists of relevant reviews will be searched for additional trials. Language restrictions will not be applied. Two reviewers will independently screen study eligibility and extract data. Methodological quality will be assessed according to the revised tool for the Quality Assessment of Diagnostic Accuracy Studies (QUADAS-2). Two authors will apply the QUADAS-2 assessment to all the included studies, and any discrepancies will be resolved by the third author. The following test characteristics will be extracted into 2 × 2 tables for all included studies: true positives, false positives, true negatives, and false negatives. Study-specific estimates of sensitivity and specificity with 95% confidence intervals will be displayed in forest plots. When possible, we will use the bivariate random-effects model or the Rutter and Gatsonis hierarchical summary receiver operating characteristic model for statistical analysis. To investigate heterogeneity, we will include study designs, population characteristics, test characteristics, and types of reference standard as the study-level variables.

**Discussion:**

Our systematic review will allow patients, clinicians, and researchers to determine the diagnostic performance of AFP, AFP-L3/AFP, DCP, and GPC-3 for the detection of early-stage HCC and the potential roles of these diagnostic biomarkers in the existing diagnostic pathways.

Systematic Review Registration: PROSPERO 2013; CRD42013003879

## Background

### Target condition being diagnosed

The incidence of primary liver cancer has increased globally during the past two decades. Currently, liver cancer is the third highest cause of cancer-related deaths worldwide and accounts for 7% of all cancers [1]. Hepatocellular carcinoma (HCC) represents more than 90% of liver cancers, and thus, is a major contributor to global disease burden. Major risk factors for developing HCC include infections with hepatitis B virus (HBV) or hepatitis C virus, alcoholic liver disease, and nonalcoholic fatty liver disease. Most of these risk factors contribute to the development of liver cirrhosis, which is present in 80% to 90% of patients with HCC [2]. Reports have shown that the 5-year cumulative risk for the development of HCC in patients with liver cirrhosis ranges between 5% and 30% [3]. Despite advances in surveillance and available treatments, there has been little improvement in the survival rates of HCC patients; the 5-year survival rate of HCC patients remains below 12% in the United States [2].

Diagnosis of early-stage HCC followed by curative resection or liver transplantation offers the best chance for long-term patient survival. Five-year survival rates of 70% have been achieved in HCC patients with preserved hepatic function after surgical resection of single tumors less than 5 cm in diameter [4]. In addition, 5-year survival rates of more than 70% have been reported in patients with HCC meeting the Milan criteria (single nodule <5 cm or three nodules each <3 cm in diameter) after liver transplantation [4,5]. Radiofrequency ablation has resulted in 5-year survival rates of 37% in HCC patients who are not eligible for surgical resection or liver transplantation [4]. Nevertheless, survival rates of patients with advanced HCC have dropped markedly to an average survival of less than one per year [6]. Importantly, fewer than 30% of HCC patients are diagnosed early enough during surveillance to undergo surgical resection or liver transplantation [7].

### Index tests

#### Alpha-fetoprotein and lens culinaris agglutinin-reactive alpha-fetoprotein

Alpha-fetoprotein (AFP) is the most widely investigated biomarker for HCC diagnosis. Persistent elevation of AFP has been shown to be a risk factor for developing HCC and is used to help define at-risk populations [8]. However, AFP has suboptimal diagnostic performance for HCC surveillance. In patients with liver cirrhosis, fluctuating levels of AFP may reflect flare-ups of viral hepatitis, exacerbation of underlying liver disease, or HCC development [9]. Additionally, only 10% to 20% of early-stage HCC patients have abnormal AFP serum levels. Recently, this small proportion of tumors has been associated with a molecular subclass of aggressive HCC (S2 class, EpCAM positive) [10-12]. AFP levels at a cutoff value of 20 ng/ml demonstrate good sensitivity but low specificity, whereas a cutoff value of 200 ng/ml provides high specificity but a marked loss in sensitivity [13].

Lens culinaris agglutinin-reactive AFP (AFP-L3) is the glycosylated subfraction of AFP and is more specific to malignant hepatocytes than AFP [14]. Therefore, it may be useful in distinguishing between elevations in AFP due to benign conditions and HCC. AFP-L3, reported as the ratio of AFP-L3 to total AFP (AFP-L3/AFP), of more than 10% has been used as the cutoff value for HCC diagnosis [15]. However, the sensitivity of AFP-L3/AFP is low in cases where AFP is not markedly elevated [16]. Recently, highly sensitive AFP-L3 assay has been evaluated in patients with an AFP level of <20 ng/ml [17]. The diagnostic sensitivity and specificity of highly sensitive AFP-L3 assay at a cutoff level of 5% were 41.5% and 85.1%, respectively. In addition, many studies have investigated the role of AFP-L3/AFP, alone or in combination with AFP and/or des-gamma carboxyprothrombin (DCP), as a screening marker for HCC [15,18-20]. The sensitivity of AFP-L3/AFP has been shown to vary with tumor size [21].

#### DCP

DCP, also known as prothrombin induced by vitamin K absence II (PIVKA II), has been widely used as a serological marker for HCC detection over the last two decades. In 1984, Liebmann and colleagues reported for the first time the association of HCC with elevated serum DCP levels based on the use of a competitive radioimmunoassay with a DCP polyclonal antibody [22]. Subsequently, a monoclonal antibody enzyme immunoassay (EIA) was developed to quantify plasma DCP levels. Several studies reported that DCP levels were elevated in patients with HCC with the use of this monoclonal antibody EIA at a cutoff value of 0.1 absorbance units (AU)/ml [23]. Currently, EIA (Eitest PIVKA-II; Eisai, Tokyo, Japan) and electrochemiluminescence (Picolumi PIVKA-II, Eisai) kits with greater sensitivity have been developed for clinical screening of patients with small HCCs [24,25]. Mita and colleagues showed that determination of DCP levels using the more sensitive EIA method at a cutoff value of 40 mAU/ml had a moderate sensitivity (61.5%) and a high specificity (94.7%) for diagnosing HCC in high-risk populations [26]. Because elevated DCP levels may not be associated with increased AFP or AFP-L3/AFP levels in HCC patients, many studies have demonstrated that a combination of these markers has a greater sensitivity in diagnosing HCC [27-29]. The Japanese Evidence-based Clinical Practice Guidelines and Consensus-based Clinical Practice Manual recommended simultaneous measurement of DCP and AFP (or AFP-L3/AFP) for screening HCC in high-risk populations and detecting single small HCCs with high sensitivity and specificity [30].

#### Glypican-3

Glypican-3 (GPC-3) is a heparin sulfate proteoglycan that interacts with several growth factors by binding to the cell membrane via glycosylphosphatidylinositol anchors [31,32]. Because GPC-3 has only been detected in HCC cells and not in benign liver tissues, it has been investigated as a potential biomarker for the diagnosis of early-stage HCC [33,34]. Recent studies demonstrated that serum GPC-3 levels were higher than 300 ng/L in 50% of early-stage HCC patients with serum AFP levels of <100 μg/L [35]. Serum GPC-3 levels at a cutoff value of 300 ng/L had a sensitivity and specificity for HCC diagnosis of 47.0% and 93.5%, respectively [35]. Additionally, the diagnostic performance of GPC-3 was increased when tested in conjunction with human cervical cancer oncogene and AFP [36]. Therefore, GPC-3 may have potential as a biomarker for diagnosing early HCC and HCC screening in high-risk populations.

#### Multiple index tests

All serological biomarkers described above are involved in different pathways in hepatocarcinogenesis. In addition, it is expected that a single biomarker would not have adequate diagnostic accuracy to identify patients with early-stage HCC. Therefore, it is reasonable that a combination of these biomarkers would improve the diagnostic performance for early-stage HCC compared to single use. If available, we will investigate the diagnostic performance of these biomarkers when used singly or in combination.

### Alternative tests

Currently, serological tests and imaging can be used for HCC surveillance; however, the use of serological tests has not been recommended for routine screening of HCC in Western practice guidelines [37,38]. The most widely used imaging examination for HCC surveillance is ultrasonography (US). A recent meta-analysis showed that US surveillance in cirrhotic patients detected most HCC cases before clinical presentation with a pooled sensitivity of 94% [39]. However, US was less sensitive for detecting early-stage HCC with a pooled sensitivity of 63% [39]. There is little evidence to indicate the use of other imaging techniques including multi-detector computed tomography (CT) or dynamic contrast-enhanced magnetic resonance imaging (MRI) for HCC surveillance. Although novel genetic markers are continuously discovered and reported, they are not available for routine use in clinical practice.

### Rationale

The efficacy of surveillance methods for HCC in high-risk populations was evaluated in a randomized controlled trial of 18,816 HBV-infected patients in China. This study demonstrated that measurement of serum AFP levels and US every 6 months was associated with a 37% reduction in HCC-related mortality [40]. Current practice guidelines from the American Association for the Study of Liver Diseases (AASLD), the European Association for the Study of the Liver (EASL), and the European Organization for Research and Treatment of Cancer (EORTC) recommend the surveillance of at-risk individuals with US every 6 months [37,38]. The combination of US with AFP is not recommended for HCC surveillance because the small 6% to 8% gain in the detection rate does not balance the increase in false positive results and the cost of early-stage HCC diagnosis in Western countries [38]. Furthermore, US has suboptimal sensitivity for detecting early-stage HCC. Thus, it is warranted for serological tests to help identify patients with early-stage HCC that will have better survival following curative treatment (resection, liver transplantation, or ablation). Recently, several serological tests including AFP-L3/AFP, DCP, and GPC-3 have been widely investigated as diagnostic markers for early-stage HCC in at-risk populations. In this systematic review, we aim to determine the diagnostic performance of AFP, AFP-L3/AFP, DCP, and GPC-3 for HCC detection, particularly early-stage tumors meeting the Milan criteria (single nodule <5 cm or three nodules each <3 cm in diameter). Identifying the potential role of these new diagnostic biomarkers in the existing diagnostic pathways will be useful in designing future studies to evaluate the accuracy of diagnostic tests and to understand study results.

### Objectives

Our primary objective is to determine and compare the diagnostic performance of AFP, AFP-L3/AFP, DCP, and GPC-3, either singly or in combination, for early-stage HCC diagnosis. Our secondary objective is to evaluate the potential role of these new diagnostic biomarkers, either singly or in combination, in the existing diagnostic pathways [41].

## Methods/design

### Criteria for considering studies for this review

#### Types of studies

We will include cross-sectional studies that consecutively or randomly recruit target populations. The index tests and reference standards should ideally be performed on all patients. We will not include diagnostic case–control studies because such studies are likely to overestimate diagnostic performance [42]. In addition, the measures of accuracy may vary with the prevalence and stage-distribution of the target condition [43].

#### Participants

High-risk populations are defined as adult patients in whom HCC surveillance is recommended by the clinical practice guidelines of AASLD, EASL, and EORTC. According to AASLD guidelines, populations at high risk of HCC include Asian male HBV carriers over age 40 years; Asian female HBV carriers over age 50 years; HBV carriers with a family history of HCC; African/North American Blacks with hepatitis B; cirrhotic HBV carriers; patients with hepatitis C cirrhosis; patients with stage 4 primary biliary cirrhosis; and patients with cirrhosis due to genetic hemochromatosis, alpha 1-antitrypsin deficiency, or other etiologies. According to EASL guidelines, high-risk populations are cirrhotic patients, non-cirrhotic HBV carriers with active hepatitis or family history of HCC, and non-cirrhotic patients with chronic hepatitis C and advanced liver fibrosis F3. The diagnosis of liver cirrhosis and chronic viral hepatitis in primary studies will be assessed. Exclusion criteria are defined as primary studies that mainly recruit low-risk populations (healthy populations or participants without any predisposing factors for developing HCC).

#### Index tests

The index tests include AFP, AFP-L3/AFP, DCP, and GPC-3 as described previously in the Background section.

#### Comparator tests

Not applicable.

#### Target conditions

The target condition will be proven HCC.

#### Reference standards

Studies will be eligible for this review if clinical diagnostic criteria recommended by the AASLD or EASL-EORTC were used as the primary reference standards. The pathological diagnosis of HCC is recommended for all nodules occurring in non-cirrhotic livers and for those cases with inconclusive non-invasive diagnosis of HCC in cirrhotic livers. In cirrhotic patients, non-invasive diagnosis of HCC is established when one imaging technique for nodules >2 cm and two imaging techniques (multi-detector CT and dynamic contrast-enhanced MRI) for nodules 1 cm to 2 cm in diameter reveal HCC radiological hallmarks (arterial hypervascularity and venous/late phase washout). Because clinical practice guidelines are routinely updated, we will use the criteria of reference standards that the primary studies adopted.

### Search methods for identification of studies

#### Electronic searches

We will search the following databases: CENTRAL (the Cochrane Library, latest issue February 2013), Medline (January 1950 to February 2013), Embase (January 1980 to February 2013), Science Citation Index (January 1981 to February 2013), and the Chinese National Knowledge Infrastructure (January 1997 to February 2013). We will also search the Meta-analyses *van Diagnostisch Onderzoek* (MEDION) and Aggressive Research Intelligence Facility (ARIF) databases to identify diagnostic systematic reviews that include primary studies. Language restrictions will not be applied. The searches will be refined using the Boolean term “AND” between the topics of HCC and index tests (AFP, AFP-L3/AFP, DCP, and GPC-3). The details of the Medline database search are provided below.

### Searching strategies of the Medline database

1. Carcinoma, Hepatocellular

2. hepatocellular Carcinoma.mp.

3. exp Liver Neoplasms/

4. malignant hepatoma.mp.

5. hepatoma.mp.

6. liver cancer.mp.

7. liver tumor.mp.

8. liver tumour.mp.

9. hepatic cancer.mp.

10. cancer of liver.mp.

11. hepatic tumor.mp.

12. hepatic tumour.mp.

13. 1 or 2 or 3 or 4 or 5 or 6 or 7 or 8 or 9 or 10 or 11 or 12

14. alpha-Fetoproteins/

15. alpha-fetoprotein.mp.

16. alpha fetoprotein.mp.

17. AFP.mp.

18. alpha-1-fetoprotein.mp.

19. alpha1fetoprotein.mp.

20. alpha 1 fetoprotein.mp.

21. alpha-fetoglobulin.mp.

22. alpha fetoglobulin.mp.

23. 14 or 15 or 16 or 17 or 18 or 19 or 20 or 21 or 22

24. AFP-L3.mp.

25. AFPL3.mp.

26. AFP L3.mp.

27. Lens culinaris agglutinin-reactive alpha-fetoprotein.mp.

28. lectin-bound AFP.mp.

29. L3 fraction.mp.

30. L3-fraction.mp.

31. glycosylated AFP.mp.

32. frucosylated AFP.mp.

33. 24 or 25 or 26 or 27 or 28 or 29 or 30 or 31 or 32

34. DCP.mp.

35. Des-gamma carboxyprothrombin.mp.

36. PIVKA*.mp.

37. PIVKA-II.mp.

38. PIVKA II.mp.

39. Protein induced by vitamin K absence*.mp.

40. 34 or 35 or 36 or 37 or 38 or 39

41. Glypicans/

42. Glypican-3.mp.

43. Glypican3.mp.

44. Glypican 3.mp.

45. GPC3.mp.

46. GPC-3.mp.

47. GPC 3.mp.

48. 41 or 42 or 43 or 44 or 45 or 46 or 47

49. 23 or 33 or 40 or 48

50. 13 and 49

#### Searching other resources

Reference lists of relevant reviews will be searched for additional trials.

### Data collection and analysis

#### Selection of studies

Two authors (TSH, YCS) will independently screen titles and abstracts to identify potentially relevant studies. After the screening, we will retrieve full texts of potentially eligible studies to assess whether the individual studies fulfill the inclusion criteria. Disagreements will be resolved by the third author (HYC).

#### Data extraction and management

We will extract the following information into the prespecified data extraction form:

1. Study characteristics (authors, year of publication, study designs, settings, locations, and patient enrollment strategies)

2. Test characteristics (test types, test conditions, prespecified defined cutoff values, sampling protocol, and criteria for test positivity)

3. Reference standards (histopathological diagnosis or non-invasive criteria and versions of the guideline used)

4. Participant characteristics (age, gender, ethnicity, disease types, tumor sizes, and tumor stages)

5. Statistics for meta-analysis (true positives (TP), false positives (FP), true negatives (TN), and false negatives (FN))

For test statistics, we will construct 2 × 2 tables to facilitate meta-analysis of the summary estimates of sensitivity and specificity. If these direct data are lacking in the articles, we will try to reconstruct the 2 × 2 table using the aforementioned publication information.

#### Assessment of methodological quality

Methodological quality will be assessed according to the revised tool for QUADAS-2 [44]. The full QUADAS-2 tool consists of four domains: patient selection, index test, reference standard, and flow and timing. Each domain will be assessed in terms of risk of bias according to the signaling questions, and the first three domains will also be adjudicated in terms of concerns regarding applicability. The details are as follows:

1. Patient selection

Risk of bias: Could the selection of patients have introduced bias?

Signaling question 1: Was a consecutive or random sample of patients enrolled?

Signaling question 2: Did the study avoid inappropriate exclusion?

Signaling question 3: Did the study include patients with previous imaging studies or biomarker testing?

Applicability: Were there concerns that the included patients and setting did not match the review question? If the primary studies primarily included patients with a low risk of developing HCC, it would be of high concern regarding applicability. If the primary studies included patients who fulfilled the screening recommendations of the guidelines, it would be of low concern regarding applicability.

2. Index test

Risk of bias: Could the conduct or interpretation of the index test have introduced bias?

Signaling question 1: If a threshold was used, was it prespecified?

Signaling question 2: Were the index test results interpreted without knowledge of the results of the reference standard?

Applicability: Were there concerns that the index test, its conduct, or its interpretation differed from the review question?

3. Reference standard

Risk of bias: Could the reference standard, its conduct, or its interpretations have introduced bias?

Signaling question 1: Was the reference standard likely to correctly classify the target condition?

Signaling question 2: Were the reference standard results interpreted without knowledge of the results of the index test?

Signaling question 3: Was AFP incorporated into the non-invasive criteria? (This question will be used for evaluation of the diagnostic performance of AFP and AFP-L3/AFP.)

Applicability: Were there concerns that the target condition as defined by the reference standards did not match the question?

4. Flow and timing

Risk of bias: Could the patient flow have introduced bias?

Signaling question 1: Was there an appropriate interval between the index test and reference standard?

Signaling question 2: Did all patients receive the same reference standard (histopathology or non-invasive criteria for liver cirrhosis)?

Signaling question 3: Were all patients included in the analysis?

Signaling question 4: Were all patients adequately followed up? At least 3 months for negative results of reference standards would be reasonable.

The signaling questions will be answered as yes, no, or unclear. Risk of bias and concerns regarding applicability will be rated as low, high, or unclear. Two authors (TSH, YCS) will initially test the pilot QUADAS-2 items in three studies. If poor agreement is noted, we will refine the tool content and/or coding guidelines. After reaching good agreement, we will apply the updated form to complete the QUADAS-2 assessment for all included studies. Discrepancies will be resolved by the third author (HYC).

#### Statistical analysis and data synthesis

*Descriptive analysis: this* will offer an overview of all available studies and will be presented in two separate tables stratified by the index tests. One table will include study design, participants, test characteristics, and reference standards. The other table will provide details on study quality according to the review-specific QUADAS-2 tool mentioned above. The following test characteristics will be extracted into 2 × 2 tables for all included studies: TP, FP, TN, and FN. Study-specific estimates of sensitivity and specificity with 95% confidence intervals will be displayed in forest plots using Review Manager (Version 5.2). These graphical displays will reveal the variations in accuracy among the studies and the different types and brands of the index tests.

*Inferential statistics:* we will use the bivariate random-effects model with or without covariates to obtain summary estimates of sensitivity and specificity in studies where a common cutoff value was applied for the interpretation of the index tests [45]. Otherwise, the Rutter and Gatsonis hierarchical summary receiver operating characteristic (HSROC) model will be used to investigate heterogeneity in the summary estimates of sensitivity and specificity at different cutoff levels of the index tests [46]. We will test for differences among the diagnostic tests by including tests used as covariates in the model. It is expected that most studies will use only one diagnostic test, therefore, most comparisons will be indirect. However, we may be able to investigate within study comparisons in studies that used more than one test. The results will also be displayed using SROC curves. The model fitting techniques will be performed using SAS (version 9.2) and R (version 2.15.2) software [47].

#### Investigations of heterogeneity

To investigate heterogeneity, we will include study design (prospective or retrospective and year of publication), population characteristics (gender, ethnicity, age, disease types, and stage distribution), test characteristics (cutoff value, test type, and number of tests per screening round), and versions of reference standards as our study-level variables. We will test these study-level covariates in the bivariate model in the common threshold or add them to the Rutter and Gatsonis HSROC model to evaluate heterogeneity in test threshold, diagnostic accuracy, and the shape of curves. The likelihood ratio test will be used to determine the statistical significance of the covariates included in the models.

#### Sensitivity analyses

A sensitivity analysis will be conducted to test the impact of the results according to the methodological quality items rated by the QUADAS-2 tool. The reference standards may differ slightly among the studies because earlier studies may include AFP in the non-invasive criteria to diagnose HCC. However, AFP has been removed from the latest versions of the guidelines. Thus, there should be some variation if the non-invasive criteria were used to diagnose HCC. If many studies incorporate AFP level as one of the criteria of the reference standards, we will try to extract the data without using the AFP criterion. If there are few studies, we will conduct sensitivity analysis using the AFP criterion.

#### Assessment of reporting and publication bias

Reporting and publication bias will not be assessed for two reasons. First, investigation of reporting and publication bias in diagnostic accuracy studies has been shown to be problematic because many studies are performed without study registration [48-50]. Therefore, assessment of publication and reporting bias from registration is not possible. Second, funnel plot-based approaches have been shown to be misleading for reviews of diagnostic test accuracy [48,49].

## Discussion

Our systematic review will allow patients, clinicians, and researchers to determine the diagnostic performance of AFP, AFP-L3/AFP, DCP, and GPC-3 for the detection of HCC, particularly early-stage HCC, and the potential roles of these new diagnostic biomarkers in the existing diagnostic pathways. This systematic review will also help guideline developers and policy makers to provide recommendations for the use of these serological tests in clinical practice.

### Systematic review status

The systematic review is currently in the searching and screening phase of study eligibility. We expect to complete the review by October 2013.

## Abbreviations

AASLD: American Association for the Study of Liver Diseases; AFP: Alpha-fetoprotein; AFP-L3: Lensculinaris agglutinin-reactive alpha-fetoprotein; ARIF: Aggressive Research Intelligence Facility; AU: Absorbance unit; CT: Computed tomography; DCP: Des-gamma carboxyprothrombin; EASL: European Association for the Study of the Liver; EIA: Enzyme immunoassay; EORTC: European Organization for Research and Treatment of Cancer; FN: False negatives; FP: False positives; GCP-3: Glypican-3; HBV: Hepatitis B virus; HCC: Hepatocellular carcinoma; HSROC: Hierarchical summary receiver operating characteristic; MEDION: Meta-analyses *van diagnostisch onderzoek*; MRI: Magnetic resonance imaging; PIVKA II: Prothrombin induced by vitamin K absence II; QUADAS-2: Quality assessment of diagnostic accuracy studies; TP: True positives; TN: True negatives; US: Ultrasonography.

## Competing interests

The authors declare that they have no competing interests.

## Authors’ contributions

PJC was the lead researcher of the project. All authors contributed substantially to the design, methodology, writing, and revision of the protocol. All authors have read and approved the final manuscript.
